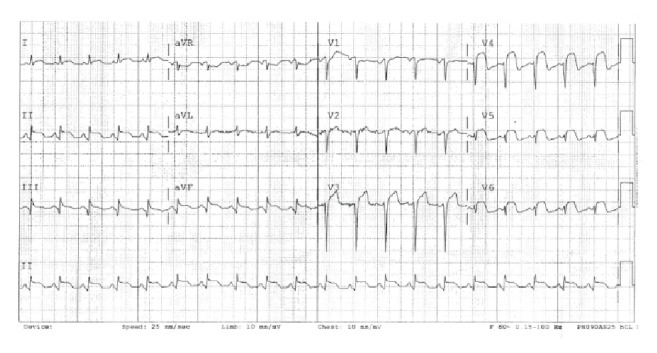# “Spice” (Synthetic Marijuana) Induced Acute Myocardial Infarction: A Case Series

**DOI:** 10.1155/2017/9252463

**Published:** 2017-07-26

**Authors:** E. Ul Haq, A. Shafiq, A. A. Khan, A. A. Awan, S. Ezad, W. J. Minteer, B. Omar

**Affiliations:** ^1^University of South Alabama, Mobile, AL, USA; ^2^Saint Luke's Mid America Heart Institute, Kansas City, MO, USA; ^3^John Hunter Hospital, Newcastle, NSW, Australia; ^4^Hattiesburg Clinic, Hattiesburg, MS, USA; ^5^Appalachian Regional Healthcare, Cardiology Department, Middlesboro, KY, USA

## Abstract

Marijuana is the most widely abused “recreational” substance in the United States, with highest prevalence in young adults. It is reported to cause ischemic strokes, hepatitis, anxiety, and psychosis. Although it is associated with dose dependent tachycardia and can lead to coronary vasospasm, it has not been directly related to acute myocardial infarction (AMI). Marijuana induced coronary vasospasm can result in endothelial denudation at the site of a vulnerable atherosclerotic plaque in response to hemodynamic stressors, potentially causing an AMI. Spice refers to herbal mixture with composition and effects similar to that of marijuana and therefore is referred to as “synthetic marijuana.” Herein, we report 3 cases of spice induced ST-segment elevation myocardial infarction. All patients were relatively young and had few or absolutely no risk factors for cardiovascular disease. All patients underwent emergent coronary angiography, with two needing stent placement and the third requiring only aspiration thrombectomy. Our case series emphasizes the importance of suspecting and investigating synthetic marijuana use in low risk young adults presenting with AMI.

## 1. Case 1

**Figure 1:**
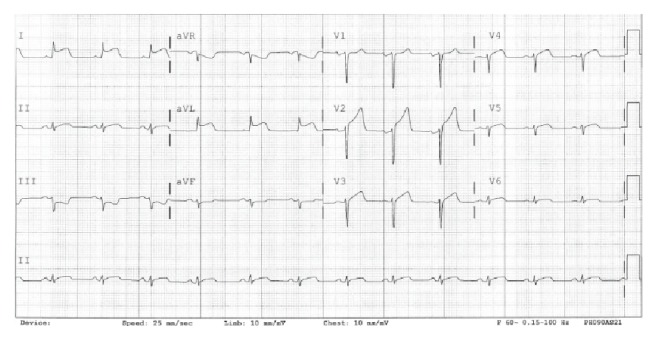


A 31-year-old African American male, with no past cardiovascular history presented to the emergency room (ER) with sharp left sided chest pain radiating down the left arm. The pain started 30–40 minutes after he smoked spice (synthetic marijuana). On physical examination, he had an elevated blood pressure of 163/89 mmHg and the electrocardiogram (EKG) revealed anterolateral ST elevations consistent with STEMI (Figure 1). The patient underwent an emergent left heart catheterization. Coronary angiography revealed normal left main coronary artery, subtotally occluded mid left anterior descending artery (LAD) with an extensive clot burden and TIMI-I flow distally. The right coronary and left circumflex arteries were widely patent. Patient had successful aspiration thrombectomy using AngioJet, followed by a bare metal stent deployment in the mid LAD. The transthoracic echocardiogram showed moderate anteroseptal hypokinesis with mild left ventricular systolic dysfunction and ejection fraction of 45–50%.

His hospital stay was uneventful and he was discharged three days later on the following medications: aspirin 81 mg daily, clopidogrel 75 mg daily, atorvastatin 40 mg at bed time, and metoprolol tartrate 25 mg twice daily. Since the patient had no risk factors for coronary artery disease, we suspected that his STEMI could be related to plaque instability caused by smoking spice.

## 2. Case 2

**Figure 2:**
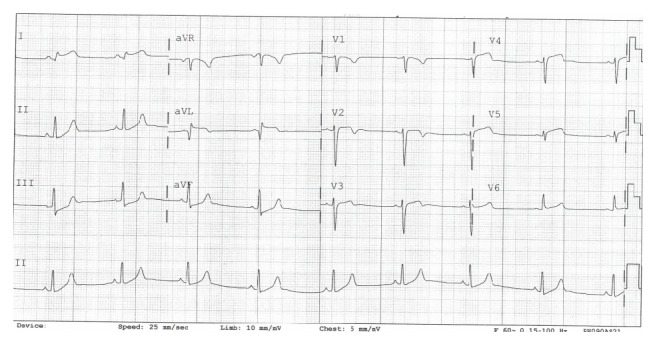


A 26-year-old Caucasian male, with past medical history of hypertension, presented to the hospital with a 2 hours' history of retrosternal nonradiating pain at rest. The patient admitted to smoking spice a few hours prior to the onset of symptoms. As the EKG showed lateral ST elevations, the patient was taken to the cardiac catheterization laboratory immediately (Figure 2). Coronary angiography showed normal left main coronary artery and an angiographically normal left circumflex as well as a normal right coronary artery.

A large thrombus burden was present in the proximal LAD involving the origin of first diagonal (D1) branch. The patient underwent manual aspiration thrombectomy with restoration of TIMI III flow. There was no significant vessel stenosis after thrombectomy, so no stent was deployed. Left ventriculography showed EF of 60 percent with anterior wall hypokinesis. STEMI protocol was followed and patient was observed in the hospital for a few days. His discharge medications included aspirin 325 mg daily, clopidogrel 75 mg daily, and lisinopril 20 mg daily. The patient was asymptomatic at 4 weeks' follow-up.

The extensive clot burden in LAD and D1 in the absence of risk factors for CAD was suggestive of spice induced coronary thrombosis.

## 3. Case 3

A 47-year-old African American male with past medical history of hypertension presented to the hospital with 3 hours of crushing substernal pain. He had no relief of his pain using acetaminophen and subsequently decided to seek medical attention. His EKG showed extensive ST-segment elevation involving inferior and anterolateral leads (Figure 3). Emergent left heart catheterization revealed normal left main coronary artery, complete thrombotic occlusion of the distal LAD, 40 percent stenosis of mid RCA, and a normal left circumflex artery. The transthoracic echocardiogram showed apical hypokinesis with LVEF of 40–45%. The patient admitted to smoking marijuana and spice 6 hours before presentation to the hospital. A drug eluting stent was deployed to distal LAD following aspiration thrombectomy. The discharge medications included aspirin 81 mg daily, clopidogrel 75 mg daily, atorvastatin 80 mg at bedtime, carvedilol 25 mg twice a day, and fosinopril 10 mg daily. The patient followed up in the Cardiology Clinic 2 weeks after discharge and was asymptomatic.

## 4. Discussion

Although marijuana has some therapeutic indications, it is mainly used as a recreational drug by young adults. Despite emerging data about possible health benefits of marijuana, it still has many adverse effects [1, 2]. Spice, which is chemically similar to marijuana, is being marketed as a safe alternative to it despite sharing many of the adverse effects of marijuana.

Marijuana is known to contain at least 60 chemical products, but the main biological effects of marijuana result from delta-9-tetrahydrocannabinol (THC) and other cannabinoids, with cannabinoid JWH-018 being the active ingredient [3]. Effects of cannabinoids are mediated through the activation of cannabinoid receptors in brain, immune system, spleen, blood vessels, and heart. Smoking marijuana also exposes the individual to particulates and gaseous material arising from the combustion of plant products. Smoking marijuana is associated with a dose dependent increase in heart rate and supine blood pressure. It can also cause postural hypotension. Tolerance to hemodynamic effects of marijuana may occur with repeated use [4]. Marijuana has been widely reported to cause ischemic strokes, hepatitis, anxiety, and psychosis.

A summary of adverse effects of marijuana is shown below.

1. Anxiety and panic, especially in naïve users.
2. Impaired attention, memory, and psychomotor performance while under the influence.
3. Increased risk of accident driving while intoxicated with cannabis, especially in conjunction with alcohol.
4. Increased risk of acute psychotic symptoms leading to self-mutilation [5].
5. A “cannabis dependence syndrome” characterized by an inability to abstain from or to control cannabis use [6].
6. Increased risk of cancers of the oral cavity, pharynx, and esophagus, as well as leukemia among offspring exposed in utero [7].
7. Impaired educational attainment in adolescents and underachievement in adults who work in jobs requiring high-level cognitive skills.
8. Adolescents with a history of poor school performance, who initiate cannabis use in the early teens, are at increased risk of using other illicit drugs and of becoming dependent on cannabis.
9. Women who continue to smoke cannabis during pregnancy may increase their risk of having a low-birth weight baby [8].

The adverse consequences of cannabis use may diminish or disappear with sustained abstinence or reduction in use. Smoking marijuana is also associated with a fivefold increase in carboxyhemoglobin concentration and a threefold higher concentration of Tar [1] when compared to smoking tobacco which results in a decreased oxygen carrying capacity and increased factor VII activity [9]. Synthetic cannabinoids appear to be 4 to 5 times more potent and appear to have more pronounced and severe cardiovascular effects than traditional marijuana [10]. Marijuana exerts its action on cardiovascular system by stimulating sympathetic nervous system. Oxygen requirement of the myocardium increases because of tachycardia induced by sympathetic nervous system. These mechanisms can potentially result in a lower angina threshold in patients with chronic atherosclerotic narrowing. Although there is a lot of interindividual variability, typical increases in heart rate with a single marijuana cigarette range from 20% to 100%, with the peak in heart rate occurring 10 to 30 minutes after starting smoking [2]. THC can also enhance atrioventricular node conduction and decrease peripheral vascular resistance. These effects have been linked to ventricular tachycardia and ventricular fibrillation in some cases [11].

Another proposed mechanism of myocardial infarction after using marijuana is the disruption of atherosclerotic plaque in response to hemodynamic stresses. Some experts have proposed endothelial denudation and platelet activation, due to coronary artery spasm and hemodynamic stress, at the site of a vulnerable plaque as the possible mechanism [2, 12]. The vasoactive and hemostatic factors released by platelets at the site of plaque rupture cause occlusive thrombus formation, resulting in acute myocardial infarction. In vitro studies have shown that cannabis may have procoagulant effects, through increased expression of glycoprotein IIb-IIIa and P-selectin on platelets [13]. Some authors have postulated an inhibitory effect of large THC concentrations on agonist-induced platelet aggregation. Others have reported elevated aggregation of platelets in the presence of THC. Whether the direct or indirect hemodynamic effects of marijuana are sufficient to cause plaque disruption is speculative, as there are no direct studies of this phenomenon available in the literature [2].

Our case series has some important health care implications. Though spice produces effects similar to cannabis, it is sometimes more potent despite it being marketed as a safe alternative to cannabis [3, 8, 14, 15]. Easy access and the misconception that spice products are natural and harmless have contributed to their popularity. Another selling point is that the chemicals used in making spice can not be detected by the usual toxicology screens. People who use spice frequently can experience withdrawal and addiction symptoms. In addition to causing various cardiovascular effects, spice can cause gastrointestinal symptoms, hallucinations, extreme anxiety, and paranoid behavior [16–19].

## 5. Conclusion

In conclusion, our case series stresses upon the importance of suspecting and inquiring about drug abuse, especially spice abuse, in low risk young adults presenting with myocardial infarction.

## Figures and Tables

**Figure 3:**